# *Ceriodaphnia dubia* as a Potential Bio-Indicator for Assessing Acute Aluminum Oxide Nanoparticle Toxicity in Fresh Water Environment

**DOI:** 10.1371/journal.pone.0074003

**Published:** 2013-09-05

**Authors:** Sunandan Pakrashi, Swayamprava Dalai, Ahmed Humayun, Sujay Chakravarty, Natarajan Chandrasekaran, Amitava Mukherjee

**Affiliations:** 1 Centre for Nanobiotechnology, VIT University, Vellore, India; 2 UGC-DAE CSR, Kalpakkam Node, Kokilamedu, India; RMIT University, Australia

## Abstract

Growing nanomaterials based consumer applications have raised concerns about their potential release into the aquatic ecosystems and the consequent toxicological impacts. So environmental monitoring of the nanomaterials in aqueous systems becomes imperative. The current study reveals the potential of *Ceriodaphnia dubia (C. dubia)* as a bio-indicator for aluminum oxide nanoparticles in a fresh water aquatic ecosystem where it occupies an important ecological niche as a primary consumer. This study aims to investigate the aluminium oxide nanoparticle induced acute toxicity on *Ceriodaphnia dubia* in a freshwater system. The bioavailability of the aluminum oxide nanoparticles has been studied with respect to their aggregation behavior in the system and correlated with the toxicity endpoints. The oxidative stress generated by the particles contributed greatly toward their toxicity. The crucial role of leached aluminium ion mediated toxicity in the later phases (48 h and 72 h) in conjunction with the effects from the nano-sized particles in the initial phases (24 h) puts forth the dynamics of nanotoxicity in the test system. The internalization of nanoparticles (both gross and systemic uptake) as substantiated through the transmission electron microscopy (TEM) and inductively coupled plasma optical emission spectral (ICP-OES) analysis was another major contributor toward acute toxicity. Concluding the present study, *Ceriodaphnia dubia* can be a promising candidate for bio-monitoring the aluminium oxide nanoparticles in a fresh water system.

## Introduction

The present scale at which the nanotechnology industry is growing, it is estimated that it will be worth nearly a trillion dollar by the year 2015 [1], optimistic forecasts suggest the production of metal oxide nanoparticles to rise from 270, 041 tons in 2012 to 1663, 168 tons by 2020 [2]. Engineered nanoparticles find application in variety of products viz. sunscreen fillers [3], bio-imaging probes [4], photovoltaic cells [5], drug delivery [6], gene delivery [7]. Alumina, a relatively inert oxide of aluminum leads the nano-material manufacture by an estimated share of 20% of the total market production [8], it has potential uses in varied fields such as abrasives [9], flooring material [10], high performance paints [11], ultrafiltration membranes [12], jet fuel [13], rocket propulsion fuel [14], heat enhancing nano fluids [15]and vaccines [16].

With increasing proliferation in the usage it is certain that aluminum oxide nanoparticles will ultimately find a way into the biosphere [17], chemtrails left by jet fuel have already raised concerns over their role increased incidences of Alzheimer disease [18]. Several relevant studies concerning aluminum oxide nanoparticles toxicity include its detrimental effects on prokaryotic microorganisms [19], microalgae species [20], aquatic cladocerans [21], earthworms [22], nematodes [23], zebra fish [24] and cell lines [25]. Despite the growing market size of aluminum oxide and its proven toxicity record we feel that there is a dearth of available literature concerning its toxicity in the aquatic environment and a significant knowledge gap exists between time dependent monitoring of bioavailability and resulting toxicity of nanoparticles [26].

Among the fresh water aquatic invertebrates, *Daphnia magna* and *Ceriodaphnia dubia* are recognized as popular choices of test organisms for eco-toxicological studies [27], ecologically they serve as vital links between the producers (algae) and secondary consumers (fish and fish larvae). In several studies *C. dubia* has been suggested to be a significantly better ecotoxicity test organism due to higher sensitivity and shorter growth span [28], [29]. The detection and monitoring of aluminium oxide contamination can be a challenging task in complex environmental conditions. The sensitivity towards aluminium oxide and a strong dose and exposure dependence along with wide ecological distribution of daphnids in fresh water aquatic systems makes it a promising candidate for bio indicator [30].

Previously we have analyzed the toxic impact of aluminium oxide and titanium dioxide nanoparticles on the freshwater bacterial and algal isolates (*Bacillus licheniformis*, *Chlorella* and *Scenedesmus* sp) under environmentally relevant conditions [31] [32] [33]. In another study on *C. dubia,* we have elaborated the toxic response towards the titanium dioxide nanoparticles and the probable mechanisms of toxicity [34]. In the current study we intend to evaluate the acute toxicity induced by aluminum oxide nanoparticles in *Ceriodaphnia dubia* in relation to the aluminum oxide bioavailability in the test environment. Further, the solubilization of the aluminum ions from aluminum oxide, reactive oxygen species (ROS) and amount of internalized aluminum oxide were accounted for observed toxic response. Direct visualization of the damaged tissue linings along the digestive tract under transmission electron microscope validated the preliminary findings. Considering the ecological niche of *C. dubia* in fresh water aquatic environment, their toxic response can further be extrapolated to analyze the comprehensive nanotoxicity impact on the aquatic ecosystem making *C. dubia* as a potential bio indicator. Thus, it is a first of its kind of report, detailing toxic response of aluminum oxide nanoparticles in terms of the dynamic nature of nanotoxicity where their bioavailability and possible solubilization has been taken into account. Importantly, this study indicates that *C.dubia* can serve as a potential bio-monitoring tool for relevant aqueous ecosystems for highly variable and complex nanoparticle mediated toxicity.

## Materials and Methods

### Isolation, Characterization and Maintenance of *Ceriodaphnia Dubia*

The fresh water samples were collected from VIT Green Lake (12°58′10′′N, 79°9′37′′E) and analyzed for the presence of planktonic crustaceans which were isolated using 5 mL pasteur pipette (Sigma Aldrich, USA) and maintained under laboratory conditions in sterilized lake water medium prepared following the procedure as described in our earlier report [19]. *Chlorella* sp at a concentration of 10^6^ cells/mL was added to supplement as the nutrition source in the medium. Subsequently the planktonic isolates were analyzed under light microscope at 10x and 40x magnifications for morphological identification and screening of the planktonic species as the organism of interest– *Ceriodaphnia dubia,* which was isolated into separate beakers containing similar lake water medium and was subcultured on a regular basis to ensure species uniformity of the subcultured colony. *Ceriodaphnia dubia* constituted ∼40% of various Daphnia species found in the ‘VIT Green Lake’ water. Thus, it was considered to be a suitable representative candidate for the study. The requisite conditions of 20°C temperature and light: dark cycle of 14∶10 were maintained in a temperature controlled chamber (I.L.E Co., India) and fluorescent light at 3000 Lux was used to maintain the light phase. The average life span of the daphnids was noted to be 20–28 days under the specified conditions.

No specific authorization was necessary for collecting samples for the study as the lake is situated inside the University campus. The experiments involved were carried out under the confinements of the laboratory and due care was exercised not to contaminate or disrupt the lake water ecosystem.

### Procurement of Nanoparticles

Aluminum oxide (Al_2_O_3_) was procured from Sigma Aldrich (St. Louis Missouri; CAS number 1344-28-1) in the form of dry powder. The suppliers product specifications are as follows - gamma phase alumina nanoparticles, particle size <50 nm, surface area 35–43 m^2^/g (BET). All other chemicals used in this study were of analytical grade. No specific information regarding surface coatings meant for stabilizing the particles were available from the manufacturer.

### Characterization of as Received Nanoparticles

Crystalline configuration of the nanoparticles was typified by X-ray Diffraction analysis (D8 Advanced X-ray Diffractometer, Burker, Germany), 250 mg of dry aluminum oxide nanoparticle was deposited in a sample holder and was scanned in the range of 10°–100° using 2.2 kW Cu anode radiation at a wavelength of 1.54 A° produced by a Ceramic X-ray tube. Scherrer formula was employed to compute the diffraction pattern and crystalline lattice geometry. The obtained data was matched with database of Joint Committee on Powder Diffraction Standards (JCPDS).

The size and shape of the procured aluminum oxide nanoparticles were analyzed and validated through transmission electron microscopy. A uniform dispersion of aluminum oxide nanoparticle of 10 µg/mL concentration was prepared in the Millipore filtered water and subjected to sonication using an ultrasonic processor with a maximum output of 130 W and frequency of 20 kHz (Sonics Corp., USA). A drop of the dispersion was taken on a lacey copper grid and dried in a dust free chamber for 4 h and was subjected to transmission electron microscopy (Philips CM12 Transmission Electron Microscope, Netherlands).

The particles were subjected to Fourier transform infrared (FT-IR) spectroscopic techniques to ascertain the surface functional groups. About 200 mg of aluminium oxide nanoparticles were mixed thoroughly with KBr and a pellet was made using a hydraulic press. The pellet was carefully transferred to the spectrophotometer (IRAffinity-1, Shimadzu, Japan) and analyzed for the presence of surface functional groups.

### Stability Analysis of Nanoparticle Dispersion

Colloidal stability of nanoparticles in dispersion is of prime importance in terms of toxicity evaluation. A detailed stability analysis for the entire test concentrations ranging from 10–120 µg/mL were carried out at the stipulated time intervals of 0, 24 and 48 h. As the mean hydrodynamic diameter (MHD) was found to be above 1000 nm, the analysis was stopped at 48 h due to low resolving capacity of the particle size analyzer for particle size above 1000 nm.

The aliquots were taken from the dispersions of 20, 40, 60, 80, 100 and 120 µg/mL at 0, 24 and 48 h intervals. The measurements were conducted for 3 min using particle size analyzer (90 plus Particle Size Analyzer, Brookhaven instruments corporations, USA). BI-DLSW software was used to compute the mean hydrodynamic diameter (MHD) corresponding to the autocorrelation function of light scatter intensity of the solvated aluminum oxide nanoparticle surface. The concentration dependent aggregation profile involving dispersions of 20 µg/mL to 120 µg/mL was studied for 48 h. The analysis was stopped at this time point because it had reached beyond 1000 nm and particle size analyzer is not sensitive to higher size ranges. Furthermore, such dimensions it is expected to behave more like bulk aluminum oxide particles.

### Bioavailability of Aluminum Oxide Nanoparticles during Exposure

Bioavailability is an important measure influencing the toxicity response of any substance. This becomes even more relevant in case of insoluble systems like aluminum oxide where the availability of the particle is dynamic in nature. This necessitates a real time analysis of available fraction of the added concentration.

About 5 mL of aliquots were collected at 0, 24, 48 and 72 h from the middle layer of the container which represents the median concentration of the system. This was digested using excess of 1 N nitric acid followed by subsequent dilutions and filtration through 0.45 µm membrane filter to ensure interference of any undigested particle. These samples were analyzed using Inductively Coupled Plasma-Optical Emission Spectrometry (ICP-OES, Perkin Elmer Optima 5300 DV, USA). This experiment was carried out in triplicates and repeated thrice to ensure reproducibility of the results obtained.

### Toxicity Analysis

In accordance with the OECD test guidelines 202 [35], acute toxicity tests of prolonged test duration72 h were carried out. Experimental setup consisted of 100 mL glass beakers each containing 5 healthy, lab bred *C. dubia* not more than 24 h old dispersed in about 20 mL of sterile lake water having aluminum oxide nanoparticles in increasing concentrations of 20, 40, 60, 80, 100 and 120 µg/mL respectively. All the experiments were conducted in triplicates and repeated more than three times to ensure reproducibility. Data have been presented as a relative survival considering untreated group as 100%.

*C. dubia* in batches of five organisms each was taken in each glass beaker containing the sterile lake water. At the end of24, 48 and 72 h, immobile organisms were isolated under compound microscope. As per the Annex 201 of OECD 211 protocol [30] an inanimate test organism if unresponsive to physical stimulus for more 15 seconds was considered dead. Thus the toxicity endpoints expressed as LC_50_ were calculated. Throughout the toxicity analysis the mortality in untreated group was found to be less than 10%.

### Oxidative Stress Analysis

Though Reactive Oxygen Species (ROS) produced as a byproduct of normal cell metabolism, in stressful conditions it may lead to excessive production leading to extensive cell membrane damage [36], [37]. Intracellular ROS concentration was determined using 2,7-dichlorofluorescin diacetate (DCFH-DA). It interacts with intracellular ROS leading to formation of a highly fluorescent complex called dichlorofluorescein, 5 µL DCFH-DA dye was added to the tissue homogenate of treated *C. dubia* and incubated at 25°C for 30 min under dark conditions. Resulting fluorescence was detected at 485 nm excitation and an emission at 532 nm using a fluorescence spectrophotometer (SL174, 191 ELICO).

### Dissolution of Soluble Aluminum during Exposure

Dispersions of aluminium oxide nanoparticles (20, 40, 60, 80, 100 and 120 µg/mL) in the filtered lake water were incubated in a shaker at room temperature. After 24,48 and 72 h intervals of incubation, the dispersion was centrifuged at 12000 rpm at 4°C for 20 min, and then filtrated through 0.1 µm and 10 kDa membrane filter with a pore size of ∼2 nm [38], [19], [39]. As the particles present in the system were well above 100 nm, it is expected that the filtration will remove all particulate aluminium oxide from the suspension. However, a particle size analysis confirmed the complete removal of aluminium oxide nanoparticles from the system. The soluble aluminium content in the suspension was measured at a wavelength of 308.22 nm using ICP-OES (Perkin Elmer Optima 5300 DV, USA).

### Gross Internalization of Aluminum Oxide Nanoparticles

Gross internalization of aluminum oxide nanoparticles into *C. dubia* was assessed after 72 h exposure. After interaction, the organisms were isolated and washed twice in Millipore filtered water to remove loosely adhering nanoparticles from the surface. The aluminum content in acid digested tissue samples was analyzed using ICP-OES (PerkinElmer 270 Optima 5300 DV, USA). The equivalent amount of aluminum oxide was calculated from the obtained metallic aluminum content.

### Systemic Uptake of Aluminum Oxide Nanoparticles

Internalization the of nanoparticles was further probed to provide an account for residual amount of aluminum oxide which remained unchanged even after 48 h depuration. After specified 72 h exposure, organisms were collected and transferred into sterile lake water and subjected to depuration step for 48 h duration. This provides sufficient time for clearance of non-internalized nanoparticles. Depurated samples were subjected to ICP-OES analysis after digesting the tissues. The equivalent concentration of aluminium oxide was computed from metallic aluminium content obtained through the analysis.

### Transmission Electron Microscopic Analysis

The tissues of *Ceriodaphnia dubia* were noted to degrade rapidly following their death. Due to this reason, live organisms were collected for microscopic examination. Highest exposure concentration of 120 µg/mL and maximum duration of 72 h was selected for microscopy due to increased probability of observing altered or damaged structures indicating toxic impact. After 72 h exposure as many as 40 organisms were collected and fixed using glutaraldehyde followed by repeated washing. These samples were then stained using toluidine blue and semi thin (0.5 µm to 1 µm) sections were prepared. Further sectioning of the zones of interest was done and ultrathin sections of 3 nm to 10 nm thickness were observed using transmission electron microscope (Philips, Netherlands).

### Statistics Applied

To ensure statistical validity of the findings, experiments were carried out in triplicates and repeated at least twice. Final figures have been computed considering all the data sets and results were presented in terms of mean and standard deviation. Data were processed using t-test at p<0.05. Graph Pad Prism (Version 6.01) was used for all the statistical analyses. Median lethal concentration was calculated using EPA Probit Analysis Program (Version 1.5).

## Results and Discussion

### Characterization of as Received Nanoparticles

**Figure 1:**
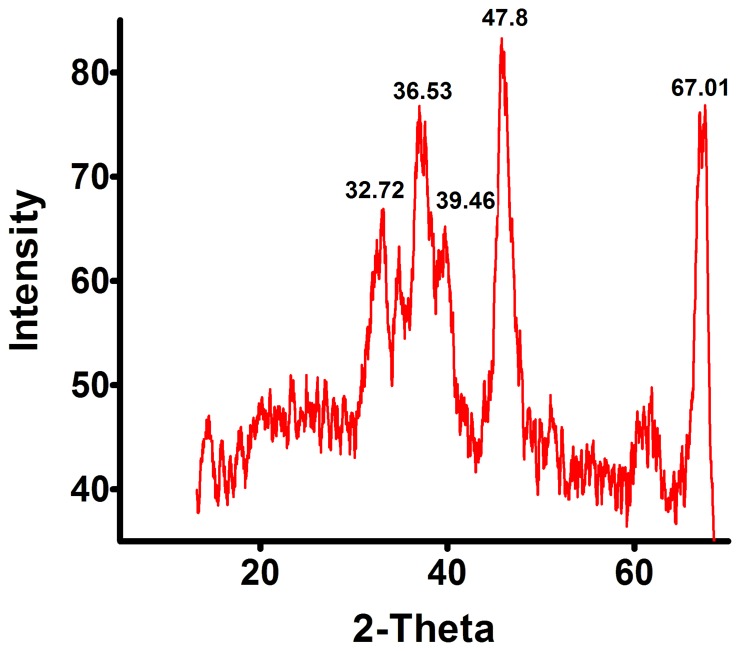
X-ray Diffraction analysis of aluminium oxide nanoparticles. The XRD results shows five dominant peaks [37.72°, 36.53°, 39.46°, 47.80° and 67.01°] confirming the crystalline nature of aluminium oxide nanoparticles.

The initial characterization of as received aluminum oxide nanoparticles confirmed the crystal structure, size and shape. The X-ray Diffraction results showed five dominant peaks [37.72, 36.53, 39.46 47.80 and 67.01° respectively], which were corroborated with the database of Joint Committee on Powder Diffraction Standards (JCPDS) card file no. 46-1215 and affirm the crystalline nature of the material to be of aluminum oxide (Figure 1).

**Figure 2:**
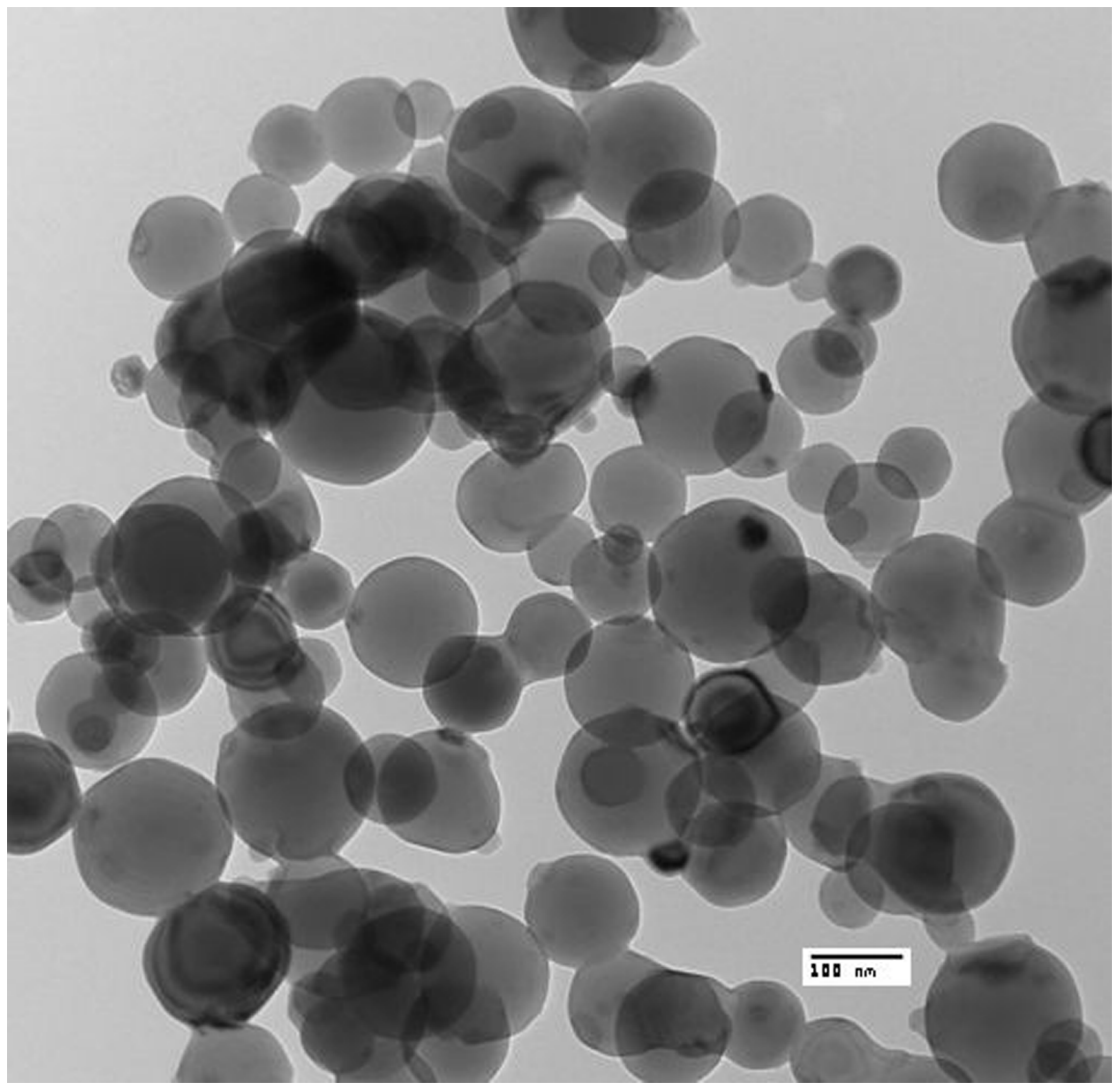
Transmission Electron Microscopic analysis of aluminium oxide nanoparticles. Transmission electron micrograph confirmed the spherical shape of the aluminium oxide nanoparticles with particle size ranging from 40 nm to 100 nm diameters. (n = 3).

The transmission electron micrographs confirmed the spherical shape of the particles with most of the particles ranging from 40 nm to 100 nm diameter. The formation of small aggregates was also noted in the TEM images (Figure 2).

The FT-IR spectroscopic analysis showed the presence of -OH stretch, -CH stretch, -CH anti-symmetrical stretch, C = O stretch and NO_2_ anti-symmetrical stretch in the as-received aluminium oxide nanoparticles. It can be assumed that majority of these functional groups were due to the stabilizing coatings present on the surface of nanoparticles. The polymer based steric stabilization of inorganic is a widely applied technique these days [40], [41]. The carboxyl, hydroxyl, amine, and ester groups are commonly found in the polymers used for stabilization [40]. In the present study, in absence of any specific data from manufacturer, we can assume that the surface groups detected through FT-IR spectroscopy is due to the presence of stabilizers on the nanoparticle surface. The presence of polymer based stabilizers can as well modulate nanoparticle-cell surface interactions such as attachment and entry of the particles in to the cellular systems [42].

### Stability Analysis of Nanoparticle Dispersion

The stability of nano-sized particles in an aqueous system is considered to be an important parameter in nano-toxicity analyses [43]. The dynamic light scattering method was employed to monitor the kinetic changes in hydrodynamic size to analyze stability of the particles in the aqueous test matrix.

At 0 h, the nanoparticles were found to have no significant differences in MHD with the varying concentrations of aluminum oxide. The MHDs for 20, 40, 60, 80, 100 and 120 µg/mL aluminum oxide concentrations were found to be 75.4±3.8, 74.2±4.6, 74.8±3.2, 76.5±3.1, 74.2±4.7 and 75.8±3.3 nm respectively.

**Figure 3:**
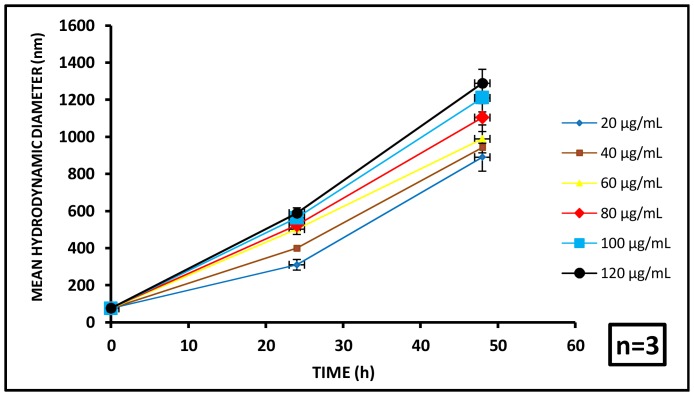
Particle aggregation profile of aluminium oxide nanoparticles. Time dependent variation in mean hydrodynamic diameter (MHD) of aluminium oxide nanoparticles was observed with respect to particle concentration. (n = 3).

At 24 h, MHDs equal to 310±21.4, 399±53.8, 502±47.2, 524±74.1, 563±49.5 and 588±67.5 nm were noted for the initial administered doses of 20, 40, 60, 80, 100 and 120 µg/mL respectively, indicating substantial aggregation of the particles. At 48 h the MHDs increased further to 890±55.1, 942±73.5, 989±71.6, 1104±52.9, 1210±81.5 and 1289±74.8 nm respectively, demonstrating further aggregation of the particles in the test medium with increasing exposure period (Figure 3).

This aggregation profile clearly demonstrates a significant increase in particle size with increasing dose and exposure period. This results in substantial loss of colloidal stability for the particles in the test system with increasing test duration. The increased MHD and consequent formation of the aggregates are expected to cause settling of particles leading to their decreased bioavailability, which may directly influence their toxicity behavior in the aqueous matrix. To confirm this apprehension the bioavailability of the aluminum oxide particles was analyzed.

The colloidal stability of nanoparticles in an aqueous test system is a prime contributory factor to its reactivity thus, leading to toxicity [44]. The size dependent toxic impact of ceria nanoparticles towards *Escherichia coli* has been reported [45]. The correlation between nanoparticle stability and toxicity has also been established in our previous reports, which showed significant toxicity of aluminium oxide nanoparticles towards the freshwater isolate, *Bacillus licheniformis* and *Cholrella* sp. in the initial 24 h when the aggregation effects were less [31], [32]. Another report on the aluminium oxide particles on sediment dwellers revealed significant correlations between the toxic impact and the particles size [46].

Uptake potential is another important aspect determining toxic impact of any nanomaterial. A variation in particle diameter giving rise to a poly-disperse system has a variable uptake frequency for each sized particles [47]. A study with fluorescent labeled polystyrene particles of varying sizes showed 100 nm particles had 2.3 folds greater uptake compared to that of 50 nm particles, 1.3 folds to that of 500 nm particles, about 1.8 folds that of 1000 nm particles. Thus, concluding that nanoparticles of 100–200 nm size acquire the optimized properties for cellular uptake. This effectively counters the misconception of smaller the size, better the uptake potential [47].

In our study, we have had a fairly monodisperse system to start with (0 h) with a polydispersity index of 0.08, but as the time progressed (beyond 24 h) the polydispersity index was measured to be 0.16 and 0.19 at 24 and 72 h respectively, indicating a significant rise in polydispersity.

Now, as concluded by [47], rather than the polydispersity, specific particle size plays major role in the uptake mechanism and the extent of internalization. The particles ranging from 100–200 nm are generally internalized through receptor mediated mechanisms; whereas, larger particles are taken up through phagocytosis [48]. In our study, the gross internalization was predominantly through oral route whereas, systemic internalization is expected to be through receptor mediated pathways during the initial exposures (<24 h) and through phagocytosis beyond 24 h. However, the dynamic state of the experiment made it difficult to comprehend the uptake process specifically.

### Bioavailability of Aluminum Oxide Nanoparticles during Exposure

**Figure 4:**
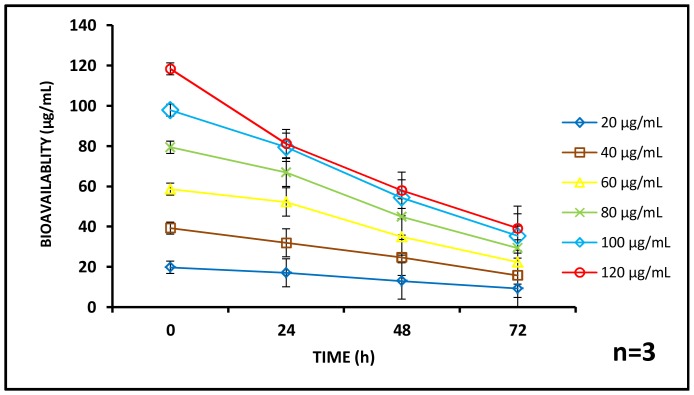
Bioavailability profile of aluminium oxide nanoparticles. The available concentration of aluminium oxide nanoparticles in the test matrix is seen to decrease with respect to time. The rate of reduction of bioavailability is faster as the concentration of the nanoparticle increases. (n = 3).

The bioavailability of aluminum oxide nanoparticles was monitored at the stipulated intervals of 24, 48 and 72 h during the exposure assessment. The available concentration of aluminum oxide was found to decrease as both exposure time and initial exposure concentrations increased. At 0 h, the available concentration of aluminum oxide was above 97.66% for administered doses of 20, 40, 60, 80,100 and 120 µg/mL respectively. Subsequently, at 24 h, bioavailable concentrations ranged between 67.66 to 85% for the same dose of administrated concentrations (20, 40, 60, 80,100 and 120 µg/mL) whereas, at 48 h, it was between 48.33 to 65%. Finally at 72 h, 32.66 to 48.5% aluminum oxide was available in the system (Figure 4). The bioavailability of the test substances is a critical element influencing toxicity and is dependent upon factors such as sorption to the vessel wall, sedimentation, aggregation and internalization within the test organism [26]. In this case we may assume that aggregation of the particles in the test medium played a crucial role in defining the bioavailability of aluminum oxide during 72 h exposure. Figure 3 clearly shows a time and dose dependent aggregation profile of the particles which possibly caused settling and consequent decreased bioavailability of the nanoparticles in the test medium. To the best of our knowledge this is the first study where bioavailability of aluminum oxide particles has been quantified to explain toxic impact in an aqueous matrix.

### Toxicity Analysis

**Figure 5:**
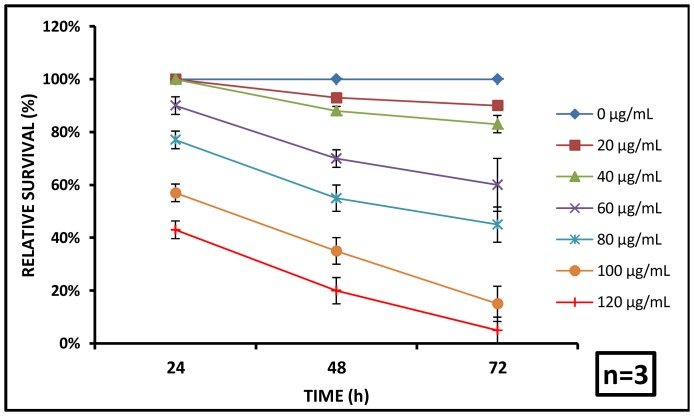
Effect of aluminium oxide nanoparticles on *C. dubia* survival. The relative survival of *C. dubia* was shown to decrease with respect to time. With nanoparticle concentration, survival rate increased which suggests reduced toxicity of nanoparticles probably due to aggregation. (n = 3).

The immobilization and subsequent mortality of *C. dubia* was dependent on exposure concentrations and time. At 24 h, all the animals were noted to be alive for 20 and 40 µg/mL exposures whereas, 90±3.33, 77±3.33, 57±3.33 and 44.34±3.33% survival was recorded upon exposure to 60, 80, 100 and 120 µg/mL dosages, respectively. At 48 h, 94.34±1.66 and 88±1.66% daphnids were alive upon 20 and 40 µg/mL exposure, whereas 70±5, 96±3.33, 35±5 and 20±5% survival were noted for the 60, 80, 100 and 120 µg/mL doses. Finally at 72 h, 90±6.66, 83.34±3.33, 60±3.33, 45±10, 15±3.33 and 5±1.5% survival was observed for the similar exposure concentrations in the range of 20–120 µg/mL respectively (Figure 5).

Furthermore, the median lethal concentrations were computed to be 117.8 µg/mL, 86.4 µg/mL and 74.3 µg/mL at 24, 48 and 72 h respectively to provide a more conventional measure of toxicity potential of aluminium oxide nanoparticles on *C. dubia*. A clear dose and exposure dependent toxicity profile was noted during the study. The sensitivity towards aluminium oxide and a strong dose and exposure dependence make daphnids a promising candidate for bio indicator in fresh water aquatic systems [30].

A number of reports exist on toxic impacts of different nanomaterials on daphnids. In our recent study on *C. dubia,* median lethal concentrations (LC_50_) of TiO_2_ nanoparticles were found to be 8 µg/mL in case of light exposure and 32 µg/mL under dark [33]. The effects of ZnO, CuO and TiO_2_ nanomaterials have been analyzed on *Daphnia maagna,* which showed higher toxicity response in case of ZnO nanoparticle (LC_50_<2 µg/mL) compared to TiO_2_ (>20 µg/mL) and CuO (>79 µg/mL) mainly due to soluble nature of ZnO [49]. Another study detailing the altered behavioral and physiological responses of *D. magna* with a major focus on hopping frequency, feeding appendage, post-abdominal curling movement, and heart rate upon exposure to TiO_2_ nanoparticles and Fullerenes showed notable anomalies [50]. Silver nanoparticles were found to have significant toxic impact during acute exposures up to 1 µg/mL; whereas, CeO_2_ nanoparticles were non-toxic up to 10 µg/mL exposure concentration [51]. The silver nanoparticle induced toxicity, uptake, accumulation and nutritional alterations on *Daphnia magna* showed considerable distortions in metabolic processes [52].

Altogether, toxicity analysis showed a clear time as well as dose dependent trend. But these data did not augur well with the aggregation and bioavailability studies demonstrating a clear decrease in the bioavailability of the nano-sized particles in the test system both with the increasing dose and the exposure time. To clarify this ambiguity, we studied the possible solubilization of aluminium ions from aluminum oxide in the later phase of the test period. Under certain conditions, aluminum oxide nanoparticle has been reported to leach labile aluminum post 24 h exposures [32]. Moreover, ionic aluminum has been reported to impart higher toxicity response compared to aluminum oxide nanoparticles [53]. Based on these assumptions, another assay was taken up to quantify the amount of ionic aluminum at specific time intervals of 24, 48 and 72 h.

### Dissolution of Soluble Aluminium during Exposure

**Figure 6:**
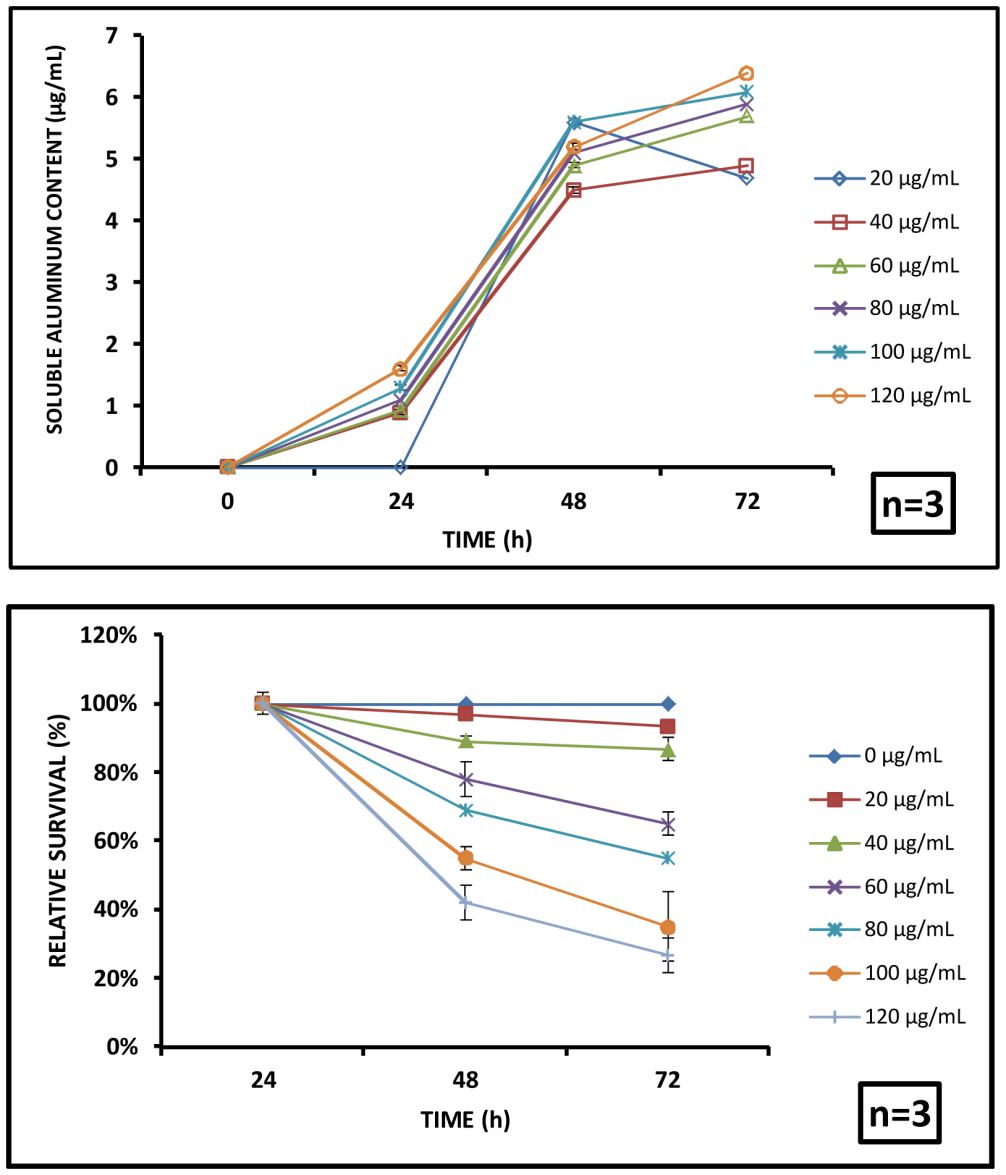
Effect of labile aluminium leached from aluminium oxide nanoparticles. (A) Dissolution of ions from the aluminium oxide nanoparticles showed an increasing trend with time, but the release kinetics is not concentration dependent. (B) The relative survival of *C. dubia* upon exposure to labile aluminium was quantified. It showed no effect up to 24 h exposure, whereas, 48 and 72 h exposures showed a significant impact on the survival compared to the untreated group.

In this study, the extent of soluble aluminum leached from the nanoparticles has been measured in the test system. During the longer exposures (at 48 h and 72 h), a significant amount of aluminum was found to have leached to the test system. At 48 h, 3.9, 4.1, 4.9, 5.1, 5.6 and 5.2 µg/mL of soluble aluminum was found in the particle free system against the corresponding exposure concentrations of 20, 40, 60, 80, 100 and 120 µg/mL respectively. At 72 h, 4.7, 4.9, 5.7, 5.9, 6.1 and 6.4 µg/mL of soluble aluminum was found in the particle free suspension against for the same initial exposure concentrations (Figure 6A). Statistical analysis validated a significant increase in soluble aluminum concentration with respect to exposure time for a particular administered concentration. However, dose dependence was not significant at all exposure concentrations when one step increment was considered.

During the initial exposure period (24 h), the inherent toxic nature of nano-sized particles played a dominant role resulting in a negative impact on the test organisms. Beyond 24 h (i.e. at 48 h and 72 h), labile aluminum leached from aluminum oxide nanoparticle also played a significant role imparting toxic effect. This is substantiated by the dissolution of aluminium ions from the aluminium oxide nanoparticles at later stages as discussed in the preceding section. A similar mechanism was proposed in our previous study with freshwater algae *Chlorella sp* where we showed an increase in the toxicity of aluminium oxide nanoparticles in spite of a significant increase in MHD of particles in the later phases. The probable mechanisms causing dissolution of aluminium oxide particles were discussed extensively and the labile aluminium was found to play the crucial role in toxicity [32]. Another report from our group ruled out any kind of ion mediated toxicity in case of titanium dioxide nanoparticles exposure to *Scenedesmus* sp. due to its insoluble nature under the test conditions [33].

The dissolution of the aluminium oxide nanoparticles in aqueous matrix is usually a two-step process involving interaction between surface and matrix leading to dissociation of Al-O complex [54].During the initial exposure up to 24 h, adsorption of natural organic matter (NOM) onto the nanoparticles due to steric or charge based attractions [55], could have prevented the release of aluminum ions. During the longer exposures of 48 and 72 h, the adsorbed NOM could induce surface reactions resulting in ligand mediated dissolution of Al^3+^ ions from the nanoparticle surface [54], which could be the reason for an increase in the released aluminum ion concentration at 48 h and 72 h.

The soluble aluminium mediated toxicity was quantified using a filtrate containing only the aluminium ions free from the traces of the nanoparticles after 24, 48 and 72 h dissolution. The suspension containing the aluminium ions leached caused no decrease in viability at 24 h even for 120 µg/mL exposure concentrations. A notable drop in the cell viability was noted after 48 h and 72 h, which was statistically significant with respect to control (Figure 6B).

### Oxidative Stress Analysis

**Figure 7:**
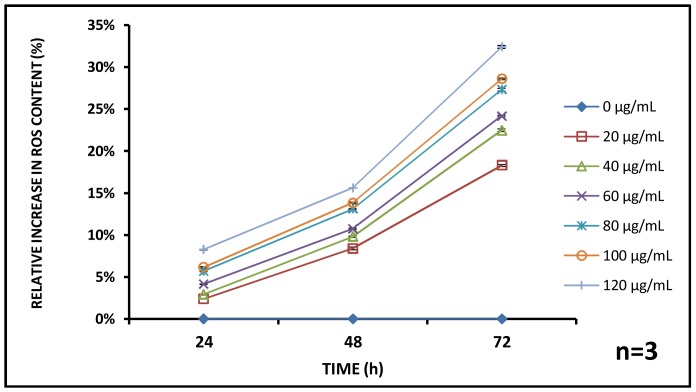
Reactive Oxygen Species profile. Generation of reactive oxygen species (ROS) was found to increase with time and with concentration suggesting oxidative stress induced toxicity of nanoparticles. (n = 3).

Oxidative stress analysis showed an overall increase in Reactive Oxygen Species (ROS) content with corresponding increment in amount of aluminum oxide nanoparticles and exposure time. At 24 h, 2.40±0.02, 2.92±0.06, 4.16±0.02, 5.71±0.09, 6.15±0.08, and 8.27±0.05; at 48 h 8.39±0.16, 9.80±0.09, 10.74±0.08, 13.10±0.04, 13.82±0.10 and 15.60±0.03% and lastly at 72 h, 18.29±1.31, 22.46±0.95, 24.16±0.14, 27.31±0.18, 28.59±1.32 and 32.40±0.07% increments in the ROS level compared to the untreated control were found for initial administered aluminum oxide at concentrations 20, 40, 60, 80, 100 and 120 µg/mL respectively (Figure 7). The role of nanomaterials inducing oxidative stress on similar species *Daphnia magna* has been identified as a major contributory factor towards cellular disruption [38]. The pathological conditions due to higher level of oxidative stress in aquatic animals has been detailed by Lushchak et al. [56].

### Gross Internalization of Aluminum Oxide Nanoparticles

**Figure 8:**
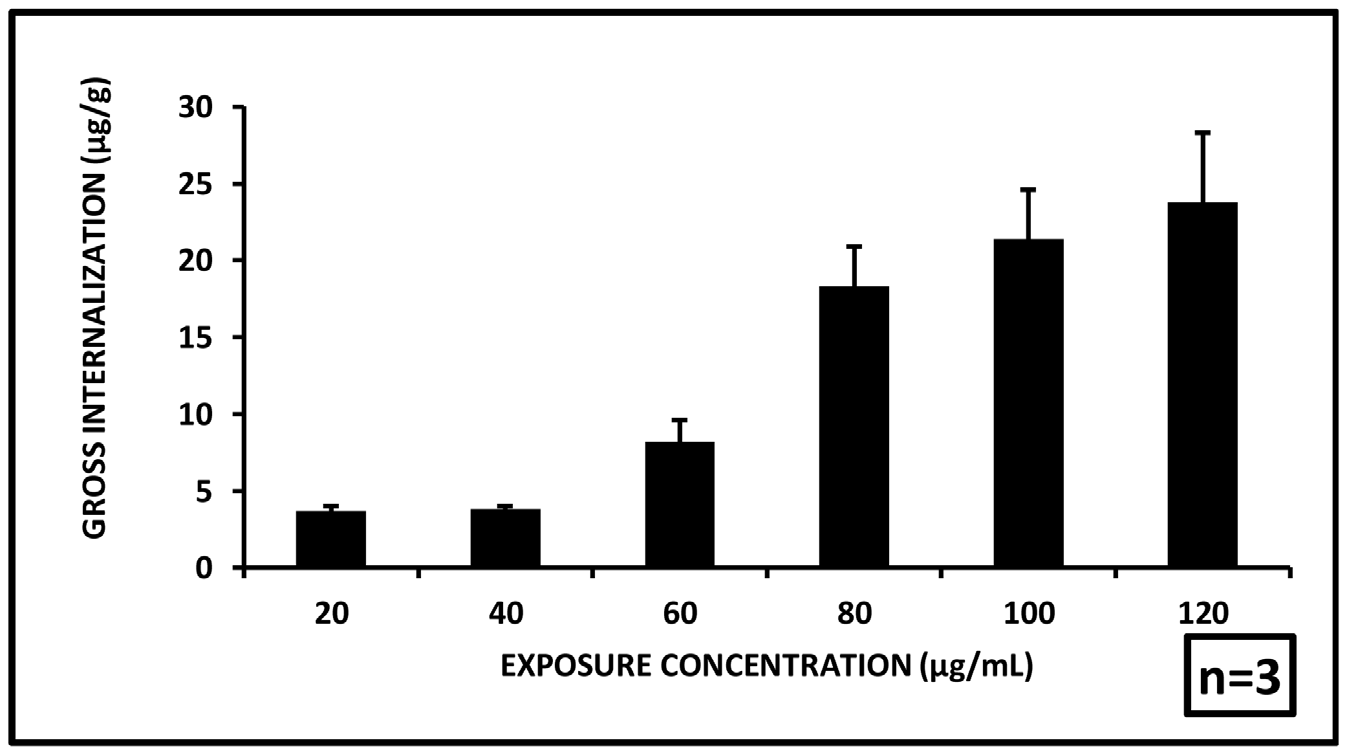
Gross internalization of nanoparticles. Nanoparticles adsorbed onto the animal outer surface and internalized into the gut were found to increase with exposure concentrations. (n = 3).

A fraction of aluminum oxide nanoparticles have been found to have been internalized into the test organisms after 72 h exposure. The internalized concentration was quantified as 3.7±0.3, 3.8±0.2, 8.2±1.4, 18.3±2.6, 21.4±3.2, and 23.8±4.5 µg/g aluminum oxide metal per gram fresh weight body weight of *C. dubia* at administered particle concentrations of 20, 40, 60, 80, 100 and 120 µg/mL respectively (Figure 8). The gross internalization data showed a strong dose dependent increase in the internalization. This indicates, the major route of entry of the nanoparticles was through the oral cavity during feeding. During the short term exposure of 72 h, the general feeding behavior did not show signs of any major disruption. The accumulation of the particles along the gut possibly caused disintegration of gut lining leading to reduced clearance which possibly resulted in the lethal systemic internalization. A few prior reports have suggested accumulation of nanoparticles (titanium dioxide) to be a major causative of disrupted clearance [57].

### Systemic Uptake of Aluminium Oxide Nanoparticles

**Figure 9:**
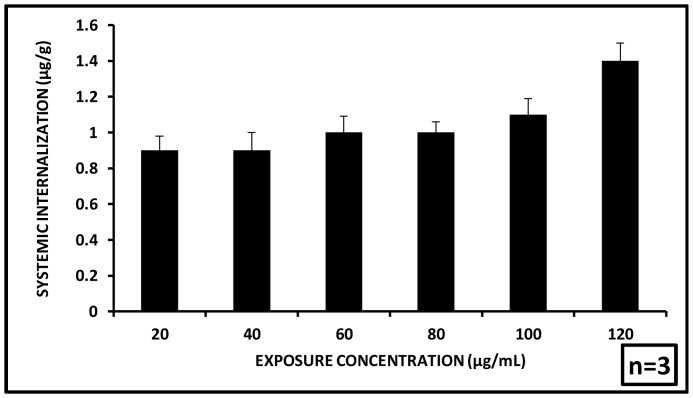
Systemic internalization of nanoparticles. Systemic internalization of nanoparticles was seen to remain almost constant till 100 µg/mL nanoparticle concentration after which a slight increase was observed for 120 µg/mL. (n = 3).

After depuration the extent of residual nanoparticle internalized to the tissue of *C. dubia* has been quantified. After 72 h exposure followed by 48 h depuration, 0.9±0.08, 0.9±0.1, 1±0.09, 1±0.06, 1.1±0.09 and 1.4±0.1 µg/g aluminum oxide per gram body weight of *C. dubia* was found to have been effectively internalized to the tissues for initial administered concentrations of 20, 40, 60, 80,100 and 120 µg/mL respectively (Figure 9). The effective internalization of nanoparticles in the tissues of *Ceriodaphnia* did show an increase with increasing exposure concentrations, a small fraction of the administered dose was found to have deposited inside the body tissues [body burden]. This might have been influenced by several factors like bioavailability, feeding, depuration pattern and disruption of the gut lining. Another major element could be a threshold limit of internalized aluminum oxide nanoparticle beyond which the organisms could not survive.

The stability analysis and the dissolution studies of aluminium oxide nanoparticles in the test medium showed that during initial exposure of 24 h, amount of the leached aluminium ions was insignificant whereas, particulate aluminium oxide was abundant. Contrastingly, during longer exposures (48 h and 72 h) the particulate aluminium oxide was less due to aggregation and subsequent settling of the particles. But there was a significant increase of leached aluminium ions in the system. Based on these observations on abundance of particles/ions, we can assume that during initial exposures the major fraction of the possible uptake would be in the form of particulate aluminium oxide; whereas, late exposures could induce uptake of aluminium ions rather than particulate aluminium oxide. However, on the basis of present analytics, it was not possible to quantify the exact proportion of particle/ion in the internalized aluminium content at any specific time point.

In our previous report, we have estimated the toxicity response of *C. dubia* towards titanium dioxide nanoparticles. A fraction of the administered nanoparticles was found to have internalized into the tissues upon similar treatment of exposure-depuration cycle. The accumulation of the particles in the cellular system contributed towards toxic impact on the organisms [32]. Additionally, the sub lethal accumulation is expected to pass on to the species preying on *C. dubia* upon feeding and will lead to bio-magnification in the food chain and will eventually pose a threat towards the ecosystem [24]. Aluminium oxide showed higher clearance ability and resultant systemic uptake was much lower compared to titanium dioxide retention. This might have resulted in a lesser toxicity response in case of aluminum oxide exposure.

### Microscopic Analysis

**Figure 10:**
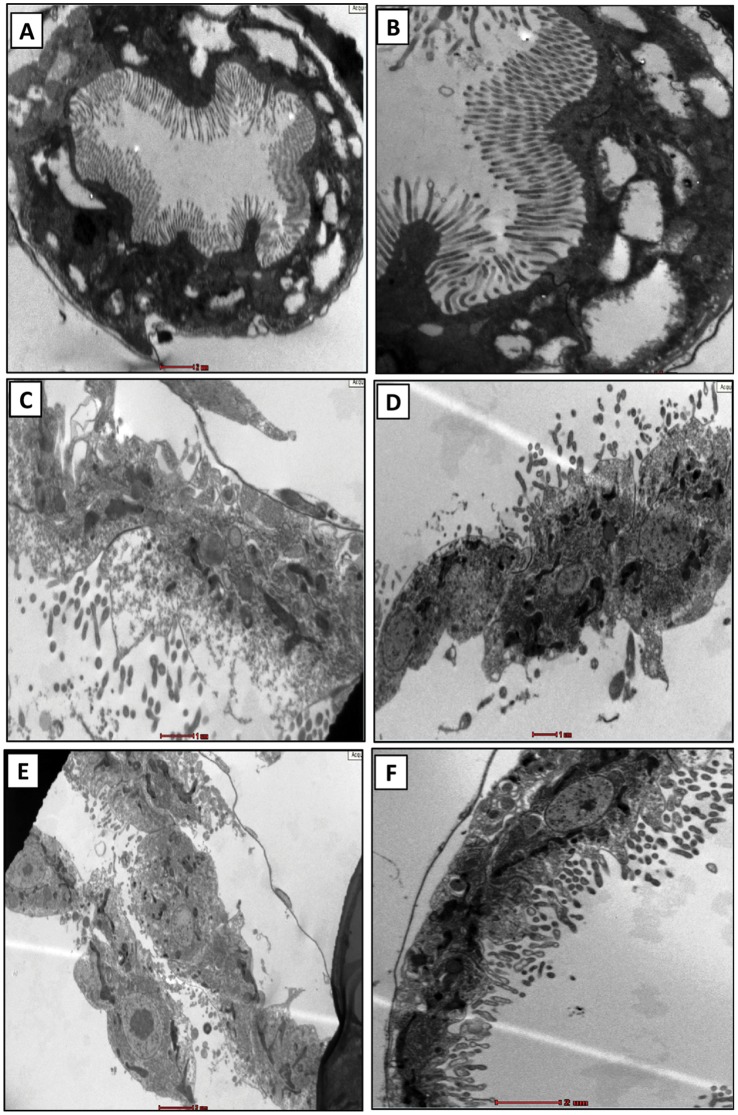
Optical microscopic analysis of *C. dubia* before and after the interaction. (A) Bright-field micrograph shows nearly transparent animal with traces of algal feed in the alimentary canal of the untreated animal. (B) Phase contrast micrograph shows natural coloration of tissues prior to the nanoparticle exposure. (C) Interacted daphinds show deposition of nanoparticle around the alimentary canal. (D) Altered coloration of the tissues can be noted in the phase contrast micrograph of interacted daphnid.

A preliminary optical microscopy (bright-field and phase contrast) was performed before and after the exposure. Before the exposure, daphnids were found to be nearly transparent with traces of algal feed in the alimentary canal. Contrastingly, after the stipulated exposure period, a large amount of aluminium oxide nanoparticle deposit was noted in and around the mid-alimentary canal (Figure 10).

**Figure 11:**
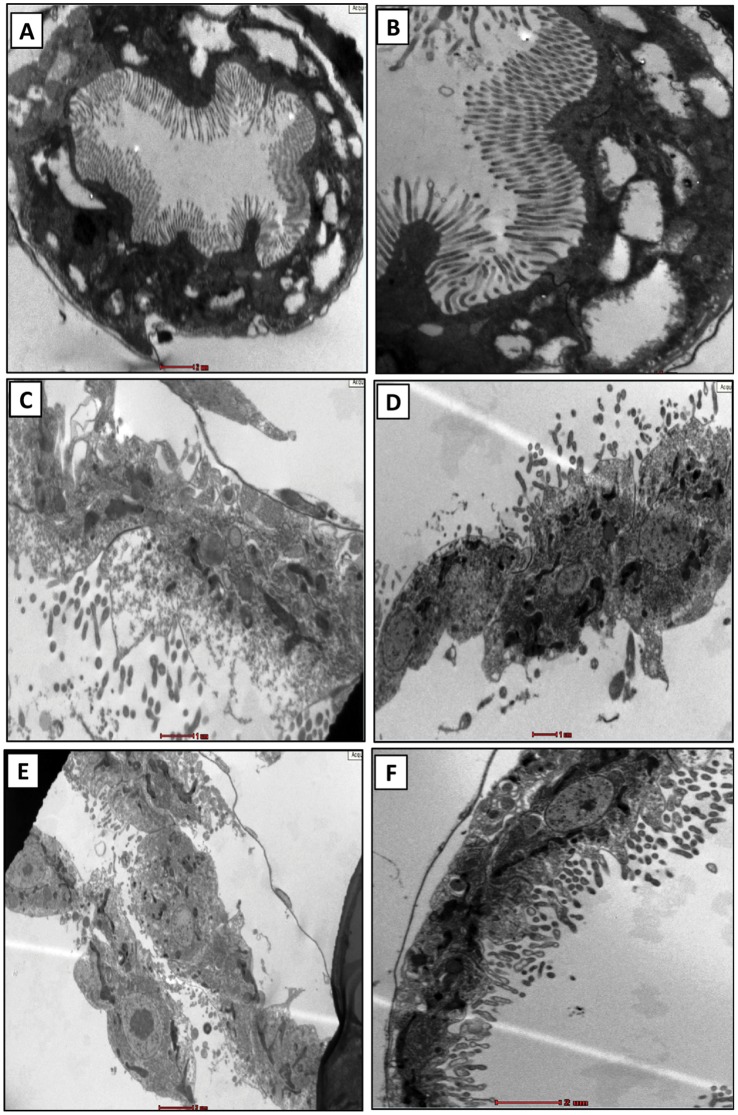
Transmission electron microscopy of the alimentary canal. Alimentary canal of untreated daphnids shows (A) uniform gut lining with intact microvilli and basal membrane; (B) a closure view of the gut lining showing healthy microvilli and basal membrane. The treated samples show (C) disrupted cellular features with (D) disintegrated micrivilli and basal membrane. Certain samples also showed (E) complete destruction of microvilli with few loosely attached cells and (F) abnormal cellular interior. BM: basal membrane; MV: microvilli. The gut lining of at least five different animals were observed to draw a conclusion (n = 5).

The alimentary canals of untreated daphnids had regular orientation of microvilli arranged on an intact basal membrane. At lower magnification, the uniformity of the whole gut lining was observed (Figure 11A). A closer view showed intact structural features of microvilli mounted on undamaged basal membrane (Figure 11B).The guts of aluminum oxide nanoparticle treated daphnids were found to have disrupted cellular features throughout the lining (Figure 11C). Higher magnifications showed disintegrated microvilli with basal membrane showing signs of damage (Figure 11D). Another area of gut showed complete destruction of the microvillus lining with a few loosely attached cells (Figure 11E). The outer portion of the basal layer at this area showed signs of disintegration whereas, inner tissues showed several abnormal cellular structures indicating an overall deleterious effect of aluminum oxide nanoparticles (Figure 11F). The findings were comprehensive and similar observations were noted throughout the samples. To ensure statistical validation of the observed features, at least three different sections of the tissues with similar features were noted during analysis. The best observation among those has been included. The findings were in close agreement with our previous study on *Ceriodaphnia* using titanium dioxide nanoparticles [32]. Another study has showed similar observations on *Daphnia magna* upon exposure to copper oxide nanoparticles [49].

## Conclusion

The acute exposure study with a strong focus on physicochemical behavior of the particles in the test medium provided a holistic perception of the toxicological response of *Ceriodaphnia dubia* towards the aluminum oxide nanoparticles. The definitive dose response in a dynamic system with time dependent binary contributory factors such as nano-size effect of the particles in the initial phase and contributions from the leached aluminum ions in the later phases was noteworthy. The toxicity data were further substantiated by the evidences of internalization of the particles from the transmission electron microscopic images that provided a clear visualization of the disruptions along the digestive tract indicating a disturbed metabolism inducing cellular death. The results suggest *C. dubia* may be a promising candidate for bio-monitoring the environmental risk of the nanomaterials in the freshwater ecosystems. Nevertheless, further detailed studies need to be conducted with other environmentally relevant nanomaterials to explore the complex dynamic relationship between the toxicity response and physical-chemical behavior of these insoluble tiny particles.
